# Draft Genome Sequence of Acinetobacter oleivorans PF1, a Diesel-Degrading and Plant-Growth-Promoting Endophytic Strain Isolated from Poplar Trees Growing on a Diesel-Contaminated Plume

**DOI:** 10.1128/genomeA.01430-14

**Published:** 2015-02-05

**Authors:** Panagiotis Gkorezis, Francois Rineau, Jonathan Van Hamme, Andrea Franzetti, Matteo Daghio, Sofie Thijs, Nele Weyens, Jaco Vangronsveld

**Affiliations:** a Centre for Environmental Sciences, Hasselt University, Diepenbeek, Belgium; b Department of Biological Sciences, Thompson Rivers University, Kamloops, British Columbia, Canada; c Department of Environmental Sciences, University of Milano–Bicocca, Milano, Italy

## Abstract

We report the 3.7-Mb draft genome of Acinetobacter oleivorans strain PF1, a hydrocarbonoclastic Gram-negative bacterium in the class Gammaproteobacteria, isolated from poplar trees growing on a diesel-contaminated plume at the Ford Motor Company site in Genk, Belgium. Strain PF1 is a potent plant-growth promoter, useful for diesel fuel phytoremediation applications.

## GENOME ANNOUNCEMENT

Acinetobacter sp. strains are known to utilize diesel fuel and other recalcitrant organics as carbon and energy sources (1). Acinetobacter oleivorans strain PF1, isolated from poplar trees growing on a diesel-contaminated plume, was shown, using GC-MS, to degrade 41% of 1.0·g·liter^−1^ (wt/vol) diesel fuel over 10 days. Partial 16S rRNA gene sequence data and phenotypic profiling indicated that PF1’s closest relative is Acinetobacter sp. DR1 (Genbank accession no. CP002080).

Genomic DNA of PF1 was extracted with a Qiagen blood and tissue kit (Qiagen NV, Hilden, Germany) prior to enzymatic digestion and ligation of sequencing adaptors using an Ion Xpress Plus fragment library kit (Life Technologies Inc., Burlington, ON, Canada). Adaptor-ligated DNA was size-selected to 480 bp on a 2% E-Gel Size Select agarose gel, and Agencourt MAPure XP beads (Beckman Coulter, Mississauga, ON, Canada) were used for purifications. An Ion library quantitation kit was used prior to amplification and enrichment with an Ion PGM Template OT2 400 kit on an Ion OneTouch 2 system. An Ion Sphere quality control kit was used to quantify the percent enriched Ion Sphere particles prior to sequencing on an IonTorrent PGM (Life Technologies Inc., Carlsbad, CA, USA) with an Ion PGM 400 sequencing kit.

In total, 2.8 million reads (mean length 303 bases) generated 853 Mb of data, of which 535,611 reads were assembled using MIRA version 3.9.9 (2) into 31 contigs, giving a consensus length of 3,766,014 bp at 43.5× coverage. Open reading frame prediction and gene annotation was carried out using RAST (3).

The complete genomes of closely related bacterial strains on the NCBI database (Acinetobacter oleivorans DR1, Acinetobacter baumanii: 13 strains, Acinetobacter pittii ANC4050, Acinetobacter calcoaceticus PHEA-2), along with sequences for Pseudomonas putida KT2440 and Escherichia coli CFT073, were aligned to the assembled PF1 contigs using Progressive MAUVE version 2.3.1 (4). The genome of PF1 consists of one circular chromosome (38.6% GC content) and includes 3,509 coding genes arranged into 668 subsystems, 5 rRNAs (5S, 16S, 23S), 56 tRNAs, and 7 noncoding RNAs.

Genes coding for the alkane hydroxylase system were found spread throughout the genome, with 9 of the 11 genes found in Pseudomonas putida Gpo1 located (5). The lower naphthalene degradation pathway is present along with the ortho-cleavage pathway, while the meta-cleavage pathway is incomplete and the lower phenanthrene degradation pathway is missing. Several gene duplicates occur for many of the biodegradative genes present. The upper naphthalene degradation pathway is incomplete as it lacks the *nahC* and *nahD* genes, although the remainder of the pathway is present leading to a complete TCA cycle.

Genes for plant-growth-promoting characteristics are present, corroborating results from phenotypic tests: 1-aminocyclopropane-1-carboxylate deaminase activity, auxin biosynthesis, siderophore production, and inorganic phosphorous solubilization.

In conclusion, Acinetobacter oleivorans PF1 is a promising candidate as an inoculant to stimulate phytoremediation of petroleum-contaminated sites.

### Nucleotide sequence accession numbers.

This whole-genome shotgun project has been deposited at DDBJ/EMBL/GenBank under the accession number JHQK00000000. The version described in this paper is version JHQK01000000.
